# Profound Alkalosis and Prolonged QT Interval Due to Inappropriate Gastrostomy Tube Loss: A Case Report

**DOI:** 10.5811/cpcem.1519

**Published:** 2024-04-30

**Authors:** Forrest Turner, Brandon Friedman, H. Pendell Meyers, Stephen W. Smith

**Affiliations:** * Carolinas Medical Center, Department of Emergency Medicine, Charlotte, North Carolina; † Hennepin Healthcare and University of Minnesota School of Medicine, Department of Emergency Medicine, Minneapolis, Minnesota

**Keywords:** *metabolic alkalosis*, *prolonged QT interval*, *case report*

## Abstract

**Introduction:**

Severe metabolic alkaloses are relatively rare but can carry a high mortality rate. Treatment involves supportive care and treatment of underlying causes.

**Case Report:**

A 55-year-old male dependent on a gastrojejunostomy tube presented to the emergency department for altered mental status. The patient had metabolic alkalosis, electrolyte abnormalities, and prolonged QT interval on electrocardiogram. Examination and history revealed that chronic drainage of gastric fluid via malfunctioning a gastrojejunostomy tube resulted in profound alkalosis. The patient recovered with supportive care, electrolyte repletion, and gastrojejunostomy tube replacement.

**Conclusion:**

This case highlights the importance of gastrointestinal acid-base pathophysiology.

Population Health Research Capsule What do we already know about this clinical entity?

*Metabolic alkalosis is frequently encountered in the emergency department. The management of severe alkalosis is well documented in the literature.*

What makes this presentation of disease reportable?

*Severe metabolic alkalosis is less commonly encountered by emergency physicians. We describe the first reported case due to inappropriate gastrostomy tube losses.*

What is the major learning point?

*This case highlights the presentation and recognition of severe metabolic alkalosis and underlying pathophysiology and describes management strategies.*

How might this improve emergency medicine practice?

*Severe metabolic alkalosis carries a high mortality. By highlighting its recognition and management, we could improve the resuscitation of these patients.*

## INTRODUCTION

Metabolic alkalosis accounts for approximately half of acid-base derangements in hospitalized patients. However, cases of severe alkalosis (pH > 7.55) are less common and carry a surprisingly high mortality.^1^^–^^4^ Anderson et al reported a mortality of 27.9% in patients with pH > 7.48, rising to 48.5% in patients with pH > 7.60.^5^ Metabolic alkalosis, which is characterized by elevated plasma pH and serum bicarbonate and decreased serum chloride, can result in elevated partial pressure of carbon dioxide (pCO_2_) via respiratory compensation. It is often associated with hypokalemia.

Metabolic alkaloses related to diuretic use or gastrointestinal losses are referred to as chloride responsive, as they are associated with the depletion of sodium chloride and volume. This results in a secondary hyperaldosteronism, thereby exacerbating potassium wasting and retention of bicarbonate within the nephron. Therapy is focused on repleting volume and electrolytes. Non-chloride responsive metabolic alkalosis has many causes including hyperaldosteronism of any etiology (eg, primary, secondary, exogenous mineralocorticoid), hypomagnesemia, hypokalemia, or exogenous alkali in the setting of renal insufficiency. Clinical manifestations of metabolic alkalosis can be non-specific and will often overlap with other associated electrolyte derangements; these include confusion, muscle cramping, tetany, seizure, cardiac dysrhythmia, and hypoventilation.^6^

## CASE REPORT

A 55-year-old male with past medical history of cerebral palsy, spastic quadriplegia, seizure disorder, and dysphagia, and dependent on a gastrojejunostomy tube presented from a skilled nursing facility with altered mental status. The patient was more lethargic and less interactive as compared to his baseline, according to staff at the facility. Otherwise, he had no communicable complaints and a limited review of systems due to lethargy. He could state his name and follow simple commands but was unable to articulate his symptoms. The patient had not been prescribed diuretic medications and had no history of recent vomiting or diarrhea. Initial vital signs included heart rate of 57 beats per minute (BPM), blood pressure 103/65 millimeters of mercury (mm Hg), respiratory rate of 12 breaths per minute, and an oxygen saturation of 93% on two liters of oxygen by nasal cannula. (The patient had no baseline oxygen requirement.)

**Image 1.**
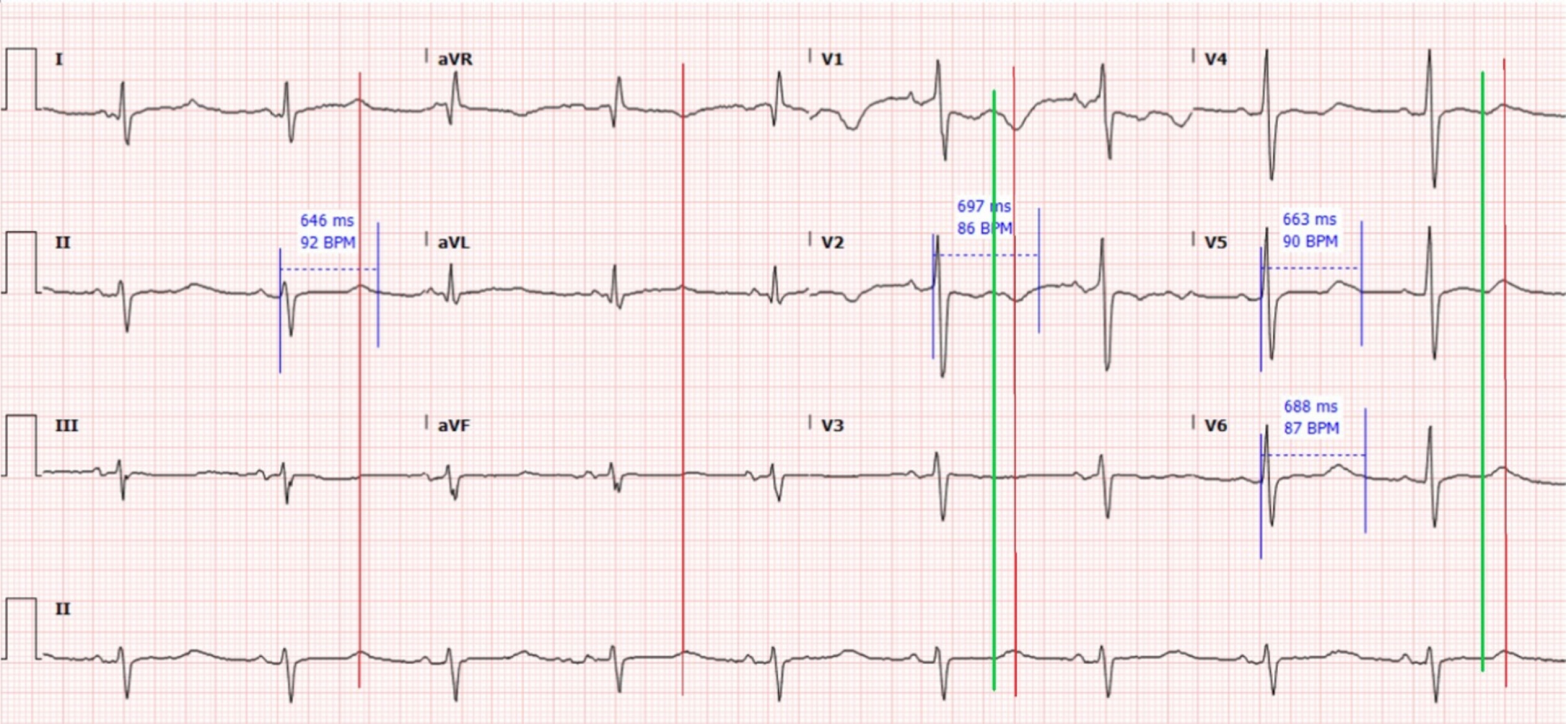
The patient’s electrocardiogram on arrival to the emergency department, with additional annotations showing sinus rhythm without obvious ischemia and extremely prolonged QT or QU interval. Several example measurements for the QU interval are shown in blue. In the precordial leads, green vertical lines mark the approximate position of the T wave, which appears flat in many leads. Red lines mark the position of the terminal component of repolarization, which are best described as U waves. *ms*, milliseconds; *BPM*, beats per minute.

Initial laboratory studies were notable for hyponatremia, hypochloremia, hypokalemia, hypocalcemia, acute kidney injury, and a serum bicarbonate above measurable range for the lab. These studies are summarized in the Table. The initial venous blood gas measured a pH of 7.61 (reference range: 7.31–7.41); pCO_2_ 77 mm Hg (35–45 mm Hg); bicarbonate 77 millimoles per liter (mmol/L) (24–28 mmol/L) and base excess 47 (−2 – +2). The initial electrocardiogram (Image 1) was notable for sinus bradycardia at a rate of 56 BPM and markedly prolonged QT or QU interval of approximately 690 milliseconds (ms), with diffuse T-wave flattening and biphasic morphology, likely followed by prominent U waves.

**Table.** Initial lab values of patient in severe metabolic alkalosis.

Serum	Patient result	Reference range
Sodium (mEq/L)	120	136–145
Chloride (mEq/L)	50	98–106
Potassium (mEq/L)	2.0	3.5–5.0
Bicarbonate (45 mmol/L)	>45^a^	20–29
Blood urea nitrogen (mg/dL)	30	8–20
Creatinine (mg/dL)	0.54^b^	0.7–1.3 (male)
Calcium (mg/dL)	4.2	8.6–10.2
Magnesium (mEq/L)	2.2	1.6–2.6

a Bicarbonate level was above the lab’s measurable range.

b Patient’s prior baseline creatinine noted to be 0.25 mg/dL.

mEq/L, milliequivalents per liter; mmol/L, millimoles per liter; mg/dL, milligrams per deciliter.

On closer inspection the gastric port of the patient’s feeding tube was noted to be draining by gravity into a Foley collection bag. The staff at the nursing facility reported that the patient’s gastric fluid was allowed to drain to gravity into a Foley bag for unclear reasons, yielding approximately 200 milliliters per day that was then discarded for an unclear number of weeks. The gastric port was disconnected from the collection bag and sealed. The patient was administered intravenous (IV) normal saline and electrolyte infusions.

**Image 2.**
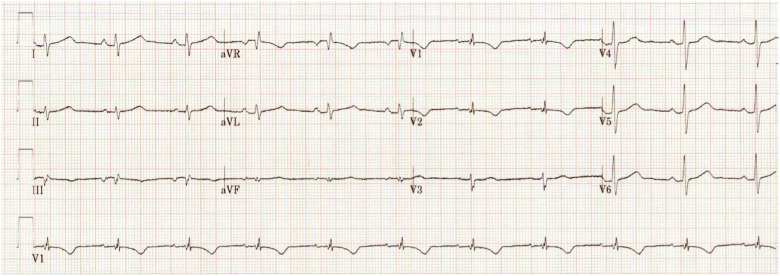
The patient’s electrocardiogram following repletion of electrolytes demonstrating improvement in QT interval.

The patient was admitted to the intensive care unit and maintained on continuous cardiac telemetry for high risk of ventricular dysrhythmias. His electrolyte derangements began to improve, and his QT interval normalized to approximately 450 ms within 24 hours, as shown in Image 2. He did not experience any significant dysrhythmias, but he did have one uncomplicated breakthrough seizure. By hospital day 2, physicians noticed intermittent dysfunction and clogging of the jejunostomy lumen of his gastrojejunostomy tube, requiring replacement by interventional radiology. Physicians surmised that feeding tube dysfunction and inability to tolerate gastric secretions likely led to symptoms that, unfortunately, inspired nursing facility staff to vent his gastric port to gravity, and the underlying cause was not addressed until hospital admission. His encephalopathy, alkalosis, hypokalemia, and other electrolyte derangements had resolved by discharge on hospital day 7.

## DISCUSSION

This case illustrates a critically ill patient with complex metabolic derangements. The patient had ongoing gastric secretion losses over a period of weeks, resulting in a slow but steady loss of volume, as well as loss of hydrogen, chloride, sodium, and potassium ions.^7^ The initial insult was maintained not only by continuous losses in the patient over time but by hypovolemia and the increasing activity of aldosterone. This resulted in further loss of potassium and impaired the kidney’s ability to excrete bicarbonate.^6^

For a patient with severe metabolic alkalosis and bradycardia, the most imminently life-threatening complication is ventricular dysrhythmias including ventricular fibrillation and polymorphic ventricular tachycardia. Prolonged QT interval is considered a risk factor for torsades de pointes (TdP), which is a form of polymorphic ventricular tachycardia preceded by long QT interval. A QT interval (or corrected QT interval when the heart rate is greater than 60 BPM) greater than 485–500 ms by the Bazett formula is considered a risk factor for the development of TdP. While the patient in this case did have a profoundly prolonged QT interval, he did not suffer any acute dysrhythmias.

Most cases of metabolic alkalosis can be adequately treated with correction of the precipitating cause and simple repletion of volume and electrolytes. In more severe or complicated cases, additional treatments with the goal of directly improving the alkalosis may be indicated. Acetazolamide inhibits carbonic anhydrase, preventing conversion of hydrogen and bicarbonate ions to carbon dioxide and water, resulting in renal loss of sodium and bicarbonate, resulting in diminution of the alkalosis. Intravenous (IV) administration of acetazolamide 500 milligrams (mg) every 12 hours has shown a small but statistically significant effect in reducing bicarbonate levels and is equivalent to more frequent dosing schedules.^8^ In patients with gastric acid loss as the cause of the alkalosis, histamine-2 receptor antagonists or proton pump inhibitors have been used as an adjunctive therapy while addressing the underlying cause.^9^ In renally impaired patients, dialysis by various methods can be used to directly lower serum bicarbonate quickly and effectively using either normal or low-bicarbonate dialysate.^10^^,^^11^

Intravenous acid solutions, most commonly hydrochloric acid, have also been used in the management of metabolic alkalosis. It is indicated when metabolic alkalosis is not correcting or not anticipated to correct with less aggressive management. Multiple case reports and case series demonstrate the safety of hydrochloric acid infusions.^12^^–^^14^ Two case reports cite chest wall necrosis resulting from hydrochloric acid infusions, highlighting the importance of administering infusions via central lines confirmed to be in good position and via the most distal port.^15^^,^^16^ Hydrochloric acid infusion dosing can be estimated by calculating the amount of hydrogen ion required via calculating the bicarbonate excess.

Bicarbonate excess can be roughly calculated by multiplying the desired decrease in plasma bicarbonate (in milliequivalents per liter [mEq/L]) by the total body water content in liters, which is roughly 60% of lean body mass in kilograms (kg) for males and 50% of lean body mass in females.^17^ Hydrochloric acid solutions with concentrations of 0.1 to 0.2 mmol/kg/hour—otherwise known as 0.1 to 0.2 normal (N) solutions—are safe formulations, with higher concentrations associated with worsened renal outcomes. A liter of 0.1 N hydrochloric acid contains 100 mEq each of hydrogen and chloride ions. Suggested maximum infusion rates include 125 mL/hour or 0.2 mEq/kg/hour. Infusions can be repeated, guided by serial electrolyte testing, and IV tubing should be changed every 12 hours due to theoretical concerns about breakdown of plastic.

Alternatives to hydrochloric acid infusions include ammonium chloride and arginine monohydrochloride, although these are both dependent on hepatic metabolism.^18^ Finally, controlled hypoventilation has been proposed as an option for critically ill intubated patients with severe alkalosis. While no data exists to suggest specific targets of minute ventilation or pCO_2_ in severe metabolic alkalosis, a few case reports mention using controlled hypoventilation to exaggerate the physiologic respiratory compensation of alkalemia in mechanically ventilated patients.^14^

Although loss of gastrointestinal secretions is one of the most common causes of metabolic alkalosis, prior case reports of severe metabolic alkalosis caused by mistakenly intentional prolonged gastrostomy tube drainage are not in the literature. Drainage or venting of a gastrostomy tube can be a temporary therapy to treat symptoms such as fullness or bloating or in cases of obstruction^19^; however, prolonged drainage of gastrointestinal secretions without addressing the underlying problem can be detrimental to the patient.

## CONCLUSION

This case, which highlights fundamental understanding of gastrointestinal and acid-base physiology, demonstrates the dangers of ongoing loss of gastric secretions and metabolic alkalosis. It serves as an example of identifying the underlying cause of alkalosis and subsequent treatment options.

## References

[1] Soifer JT Kim HT . Approach to metabolic alkalosis. Emerg Med Clin North Am. 2014;32(2):453–63. 24766943 10.1016/j.emc.2014.01.005

[2] Patel KB Espinosa J Wiley J et al . Severe alkalemia (pH 7.85): compatible with life? A triple acid-base conundrum. Mathews J Case Rep. 2016;1(2):10.

[3] Mennen M Slovid CM . Severe metabolic alkalosis in the emergency department. Ann Emerg Med. 1988;17(4):354–7. 2833137 10.1016/s0196-0644(88)80781-9

[4] Tripathy S . Extreme metabolic alkalosis in intensive care. Indian J Crit Care Med. 2009;13(4):217–20. 20436691 10.4103/0972-5229.60175 PMC2856150

[5] Anderson LE Henrich WL . Alkalemia-associated morbidity and mortality in medical and surgical patients. South Med J. 1987;80(6):729–33. 3589765 10.1097/00007611-198706000-00016

[6] DuBose T Jr . Acidosis and alkalosis. In: Harrison’s Principles of Internal Medicine, 20^th^ ed. New York, NY: McGraw Hill; 2018.

[7] Lyu HG Smink D . Stomach & duodenum. In: Current Diagnosis & Treatment: Surgery, 15^th^ ed. New York, NY: McGraw Hill; 2020.

[8] Faisy C Meziani F Planquette B et al . Effect of acetazolamide vs placebo on duration of invasive mechanical ventilation among patients with chronic obstructive pulmonary disease: a randomized clinical trial. JAMA. 2016;315(5):480–8. 26836730 10.1001/jama.2016.0019

[9] Kirsch BM Sunder-Plassmann G Schwarz C . Metabolic alkalosis in a hemodialysis patient-successful treatment with a proton pump inhibitor. Clin Nephrol. 2006;66(5):391–4. 17140170 10.5414/cnp66391

[10] Hsu SC Wang MC Liu HL et al . Extreme metabolic alkalosis treated with normal bicarbonate hemodialysis. Am J Kidney Dis. 2001;37(4):E31. 11273901 10.1016/s0272-6386(01)90017-4

[11] Ayus JC Olivero JJ Adrogué HJ . Alkalemia associated with renal failure. Correction by hemodialysis with low-bicarbonate dialysate. Arch Intern Med. 1980;140(4):513–5. 7362382

[12] Brimioulle S Vincent JL Dufaye P et al . Hydrochloric acid infusion for treatment of metabolic alkalosis: effects on acid-base balance and oxygenation. Crit Care Med. 1985;13(9):738–42. 3928258 10.1097/00003246-198509000-00009

[13] Guffey JD Haas CE Crowley A et al . Hydrochloric acid infusion for the treatment of metabolic alkalosis in surgical intensive care unit patients. Ann Pharmacother. 2018;52(6):522–6. 29359573 10.1177/1060028018754389

[14] Jones MW Williams M . Acid-base correction: a case report and review of the literature. J Intensive Care Soc. 2010;11(2):126–9.

[15] Jankauskas SJ Gursel E Antonenko DR . Chest wall necrosis secondary to hydrochloric acid use in the treatment of metabolic alkalosis. Crit Care Med. 1989;17(9):963–4. 2766771 10.1097/00003246-198909000-00023

[16] Buchanan IB Campbell BT Peck MD et al . Chest wall necrosis and death secondary to hydrochloric acid infusion for metabolic alkalosis. South Med J. 2005;98(8):822–4. 16144181 10.1097/01.smj.0000172781.27664.87

[17] Mehta A Emmett M . Treatment of metabolic alkalosis. In: UpToDate. Alphen aan den Rijn, Netherlands; Wolters Kluwer: 2022. Available at: https://www.uptodate.com/contents/treatment-of-metabolic-alkalosis?search=metabolic+alkalosis&source=search_result&selectedTitle=2~150&usage_type=default&display_rank=2. Accessed June 15, 2023.

[18] Martin WJ Matzke GR . Treating severe metabolic alkalosis. Clin Pharm. 1982;1(1):42–8. 6764161

[19] Gleeson NC Hoffman MS Fiorica JV et al . Gastrostomy tubes after gynecologic oncologic surgery. Gynecol Oncol. 1994;54(1):19–22. 8020833 10.1006/gyno.1994.1159

